# A pilot study on the characterization and correlation of oropharyngeal and intestinal microbiota in children with type 1 diabetes mellitus

**DOI:** 10.3389/fped.2024.1382466

**Published:** 2024-06-13

**Authors:** Limin Wang, Chao Gong, Ruiye Wang, Jinxue Wang, Zhanshuang Yang, Xianhe Wang

**Affiliations:** ^1^ College of Clinical Medicine, Jiamusi University, Jiamusi, China; ^2^ College of Rehabilitation Medicine, Jiamusi University, Jiamusi, China; ^3^ Jiamusi University Affiliated No. 1 Hospital, Jiamusi, China

**Keywords:** T1DM, oropharyngeal flora, intestinal flora, microbial diversity, children

## Abstract

**Background:**

Type 1 Diabetes Mellitus (T1DM) is one of the most common endocrine disorders of childhood and adolescence, showing a rapidly increasing prevalence worldwide. A study indicated that the composition of the oropharyngeal and gut microbiota changed in T1DM. However, no studies have yet associated the changes between the microbiomes of the oropharyngeal and intestinal sites, nor between the flora and clinical indicators. In this study, we examined the composition and characteristics of oropharyngeal and intestinal flora in patients with T1DM in compared to healthy children. We identified correlations between oropharyngeal and intestinal flora and evaluated their association with clinical laboratory tests in patients with T1DM.

**Methods:**

The oropharyngeal and fecal samples from 13 T1DM and 20 healthy children were analyzed by high-throughput sequencing of the V3–V4 region of 16S rRNA. The associations between microbes and microorganisms in oropharyngeal and fecal ecological niches, as well as the correlation between these and clinical indicators were further analyzed.

**Results:**

It was revealed that T1DM children had distinct microbiological characteristics, and the dominant oropharyngeal microbiota genus included Streptococcus, Prevotella, Leptotrichia, and Neisseria; that of intestinal microbiota included Blautia, Fusicatenibacter, Bacteroides, and Eubacterium_hallii_group. Furthermore, oropharyngeal Staphylococcus was significantly positively correlated with intestinal norank_f__Ruminococcaceae and Ruminococcus_torques_group in TIDM children. Moreover, in these children, differential genes in oropharyngeal and intestinal samples were enriched in metabolic pathways such as amino acid generation, fatty acid metabolism, and nucleotide sugar biosynthesis. Additionally, correlation analysis between the oropharyngeal/intestinal microbiome with laboratory tests showed significant correlations between several bacterial taxa in the oropharynx and intestines and glycated hemoglobin and C-peptide.

**Conclusion:**

Unique microbial characteristics were found in the oropharynx and intestine in children with T1DM compared to healthy children. Positive correlations were found between changes in the relative abundance of oropharyngeal and gut microbiota in children with T1DM. Associations between the oropharyngeal/intestinal microbiota and laboratory investigations in children with T1DM suggest that the composition of the oropharyngeal and intestinal flora in children with T1DM may have some impact on glycemic control.

## 1 Introduction

Type 1 diabetes mellitus (T1DM) is a chronic disease caused by the cell-mediated autoimmune destruction of pancreatic β-cells, resulting in absolute insulin deficiency. T1DM is one of the most common endocrine disorders in children and adolescents, with rapidly increasing prevalence worldwide (1). The increasing T1DM prevalence and its development in only < 10% of genetically predisposed individuals suggests that environmental factors significantly contribute to its pathogenesis (2).

The oral microbiota has been considered a possible environmental risk factor, and studies have found a strong association between oral microbiota dysbiosis and diabetes mellitus (3). Furthermore, the oral flora of T1DM children has indicated the highest abundance genera of *Brevundimonas, Ruminococcus, Micrococcaceae-unclassified, Blautia*, and *Faecalibacterium* (4). Moreover, different parts of the oral cavity are composed of different microbiota (5), and the relationship between gingival surface and salivary microbial characteristics and T1DM has also been comprehensively studied. However, the relationship between oropharyngeal microbial characteristics and T1DM has not been studied; therefore, this study mainly analyzed the relationship between oropharyngeal microbial characteristics and T1DM.

Gut microbiota composition was also highlighted as a possible environmental risk factor. Different mechanisms by which the gut microbiome may influence the pathogenesis of T1DM include immune dysregulation mediated by dysregulated gut ecology, suggesting that the microbiome plays an important role in the development and maturation of the immune system as well as intestinal leakiness (6).

The oropharyngeal microbiota is closely related to the intestinal microbiota (7). In type 2 diabetes patients, *Prevotella* co-occurred in the oral cavity and gut and indicated a positive correlation between gut Proteobacteria and oral Spirochetes, suggesting that pathogens at different sites are altered during the disease (8). However, studies correlating oropharyngeal and intestinal microflora with T1DM in children have not yet been reported.

Oral and intestinal flora experience similar changes in metabolic function. Glycine betaine degradation pathway I was significantly upregulated in oral and gut flora in T2DM (8). Furthermore, numerous animal models and human studies suggest the regulatory role of the microbiome in both normal and impaired glycemic responses (9). Gut microbes modulate short-chain fatty acid production, metabolism of bile acid, and regulation of adipose tissue to influence host glycaemic control (10–12). Unfortunately, how the microbiome controls the blood glucose in children with T1DM remains undetermined.

In this study, we analyzed the oropharyngeal and intestinal microbial profiles of children with T1DM. Correlation of alterations in oropharyngeal and gut microbiota with T1DM in children and their association with clinical phenotype. Furthermore, the study provides an important theoretical basis for exploring the effects of oropharyngeal and intestinal microbiota on T1DM children and further interventions. As per our knowledge, this study is the first to identify the characteristics of oropharyngeal flora and its correlation with intestinal flora in T1DM children.

## 2 Materials and methods

### 2.1 Ethics statement

The study was authorized by the Ethics Committee of Jiamusi University's First Affiliated Hospital (2022-500-91), and written informed consent was acquired from legal parents or guardians on behalf of the children before the study initiation.

### 2.2 Participants

**Figure 1:**
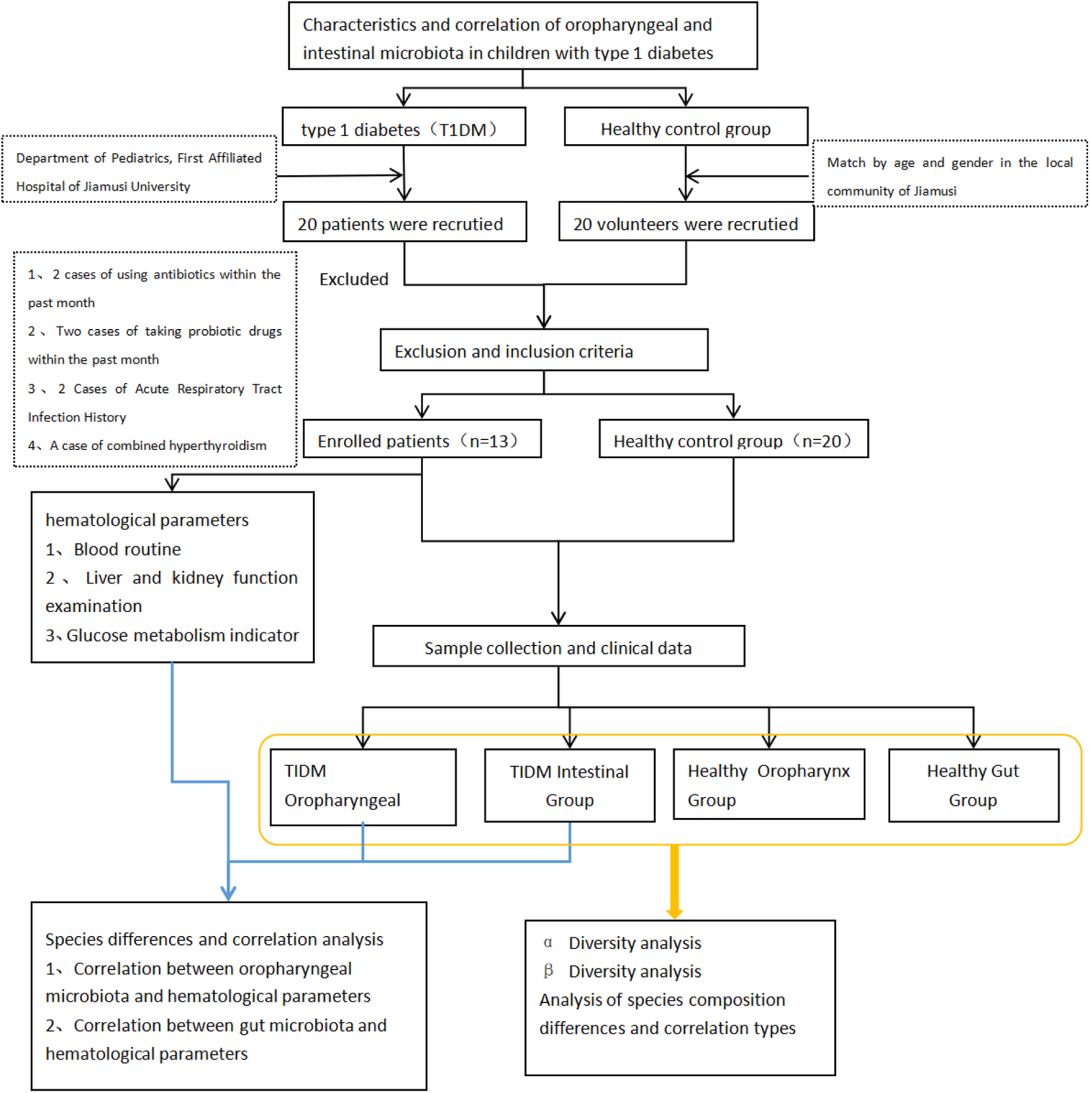
Study flowchart.

Patients with type 1 diabetes mellitus aged 2–14 years were recruited from December 2022 to June 2023 in the Department of Pediatrics of the First Affiliated Hospital of Jiamusi University. Diagnostic criteria: The diagnostic guidelines for diabetes mellitus in children, as published by the World Health Organization (WHO) in 2019, were adapted for this study. Healthy control group members were recruited from the local community of Jiamusi and matched by age and gender. Health status was defined as the absence of acute or chronic diseases and oral conditions. Children who: (1) had taken antibiotics or probiotic-containing drugs within 1 month before the experiment; (2) had combined gastrointestinal disorders such as diarrhea, constipation, and dyspepsia; (3) suffered from infectious diseases, malignant tumors, autoimmune disorders, allergies, abnormal thyroid function, and hepatic or renal insufficiency; (4) were suffering from oral diseases (such as caries, periodontal disease, oral mucosal disease, oral abscesses, fungal infections); and (5) had mental illness or other reasons for being unable to cooperate excluded from the study. Based on the above criteria, 13 children with T1DM and 20 healthy volunteers were included in this study (Figure 1).

### 2.3 Collection of clinical information

Information about gender, age, height, weight, BMI, mode of delivery, birth weight, feeding mode, age at supplementation, and family history were collected from electronic medical records. Experienced clinical staff collected 8-h fasting blood samples from the T1DM children and examined for clinical parameters, routine blood tests (SYSMEX (XN-3000), C-peptide [SIEMENS (ADVIA Centaur XP) direct chemiluminescent method], and glycated hemoglobin [CAPILLARYS (OCTA3) capillary electrophoresis method].

### 2.4 Sample collection

The subjects were asked to rinse their mouths with pure water and fast for 30 min before sampling. A tongue depressor was used to press the base of the tongue gently, and then a sterile cotton swab was rotated 10 times on the posterior wall of the pharynx and quickly placed in a 2.0 ml sterile cryogenic vial. Fecal samples were collected by directly depositing them into a fecal sampler. The stool was sampled from the center of the feces and immediately placed in 2.0 ml sterile cryogenic vials and sealed with parafilm. The kit was stored in a −80°C freezer within half an hour until sequencing analysis.

### 2.5 DNA extraction and PCR amplification

Total microbial genomic DNA was extracted from oropharyngeal swabs and stool samples using the QIAGEN MagAttract PowerSoil Pro DNA Kit (147190), per the manufacturer's instructions. The purity and the concentration of DNA were evaluated using 1.0% agarose gel electrophoresis and a Thermo Scientific Inc., USA NanoDrop® ND-2000 spectrophotometer.

### 2.6 PCR amplification

The hypervariable region V3–V4 of the bacterial 16S rRNA gene was amplified using the ABI GeneAmp® 9700 PCR thermocycler (ABI, CA, USA) with primer pairs 338F (5′-ACTCCTACGGGAGGCAGCAG-3′) and 806R (5′-GGACTACHVGGGTWTCTAAT-3′) (13). All samples were amplified in triplicate. The acquired amplified PCR product was extracted from a 2% agarose gel, purified via the AxyPrep DNA Gel Extraction Kit (Axygen Biosciences, Union City, CA, USA) per the manufacturer's guide, and then quantified using Qubit 4.0 (Thermo Fisher Scientific, USA).

### 2.7 Miseq sequencing by Illumina

Purified amplicons were pooled at equimolar concentrations and sequenced by Majorbio Bio-Pharm Technology Co. Ltd. (Shanghai, China) on the Illumina Miseq PE300 platform (Illumina, San Diego, USA) according to standard protocols for paired-end sequencing. The raw sequencing reads were uploaded to the NCBI Sequence Read Archive (SRA) database under the accession number SRP459443.

### 2.8 High-throughput sequencing data analysis

The raw FASTQ files were de-multiplexed in the Majorbio Cloud platform (https://cloud.majorbio.com) using an in-house Perl script, quality-filtered by Fastp version 0.19.6 (14), and merged by FLASH version 1.2.11 (15) with the usage criteria. Operational taxonomic unit (OTU) clustering and chimera removal were performed on the quality-controlled spliced sequences based on 97% similarity using UPARSE v11 software (16, 17). To minimize the impact of the sequencing depth on the subsequent analysis of alpha and beta diversity data, the number of sequences in all samples was rarefied to 20,000. After flattening the number of sequences, each sample's average sequence coverage (Good's coverage) could still reach 99.09%. OTU species taxonomy was annotated using the RDP classifier (18) against the Silva 16S rRNA gene database (v138) with a 70% confidence threshold. Each sample's community composition was counted at different species classification levels. 16S functional prediction analyses were performed using PICRUSt2 software (19) (version 2.2.0).

### 2.9 Statistical analysis

SPSS 26.0 software was used to analyze and compare the baseline data of the two groups. The intergroup differences for categorical data were compared using an independent two-sample t/Z test and Fisher's exact probability methods. Bioinformatics analysis of oropharyngeal swabs and fecal samples was conducted using the Majorbio Cloud platform. Based on the OTUs information, alpha diversity indices, including observed OTUs, Chao richness, Shannon index, and Simpson index, were calculated with Mothur v1.30.1 (20). The similarity among the microbial communities in different samples was determined by principal coordinate analysis (PCoA) based on bray_curtis dissimilarity using the Vegan v2.5–3 package. Furthermore, whether the differences in microbial community structure between the sample groups were significant was identified by combining PLS-DA, PERMANOVA nonparametric test, and Wilcoxon rank sum test. Moreover, using the Linear discriminant analysis (LDA) effect sizes (LEfSe) (21) the significant abundance of bacteria (phylum to genus) in the various groups was identified (LDA scores > 3, *p < 0.05*). Correlation heat maps were generated to examine the relationship between bacterial communities, groups, and clinical indicators (22).

## 3 Results

### 3.1 Study population and clinical characteristics

Gender, age, height, weight, BMI, mode of delivery, mode of feeding, age at supplementation, birth weight, and family history of T1DM children did not differ significantly from the healthy control children (*p > 0.05*). T1DM children had a smaller gestational age than healthy control children (*p < 0.05*) Table 1.

**Table 1:** Basic characteristic of diabetes group and control group [mean ± SD/*n* (%)].

Characteristics	Type 1 diabetes (*n* = 13)	Control (*n* = 20)	*P* value
Height	1.39 ± 0.19	1.32 ± 0.19	0.314
Weight	38.9 ± 16.80	33.2 ± 13.43	0.292
BMI	19.7 ± 8.05	18.06 ± 3.81	0.442
Age	9.13 ± 2.81	8.11 ± 0.49	0.333
Gestational age	37.3 ± 0.75	38.51 ± 1.52	0.013
Birth weight	3.36 ± 0.52	3.39 ± 0.63	0.868
Add supplementary food age	6 ± 0.41	6.7 ± 1.84	0.189
Gender (male)	5 (27.8)	13 (72.2)	0.169
Gender (female)	8 (53.3)	7 (46.7)	
Birth way (eutocia)	5 (62.5)	3 (37.5)	0.213
Birth way (cesarean section)	8 (32.0)	17 (68.0)	
Feeding ways (Breast milk)	13 (41.9)	18 (58.1)	0.508
Feeding ways (Mixed)	0 (0)	2 (100)	

### 3.2 Oropharyngeal microbiome composition of T1DM and healthy children

**Figure 2:**
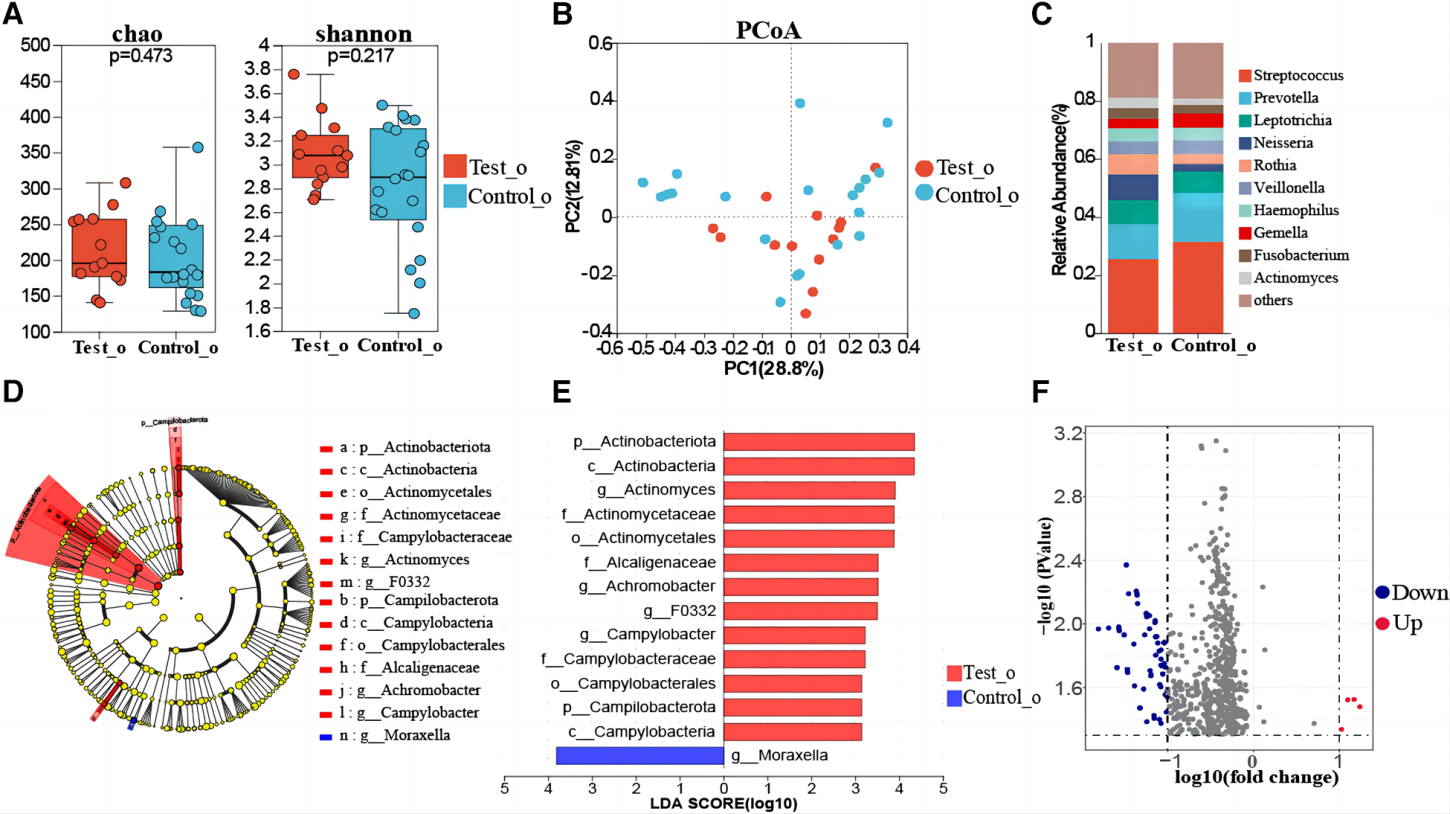
Oropharyngeal microbial composition of T1DM children. (**A**) Comparison of biome alpha diversity, the *p-value* was calculated by Wilcoxon rank sum test. (**B**) β-diversity analysis of the microbiota. PCoA based on the β-diversity of the bray-curtis. Similarity analysis (ANOSIM) assessed if each group is significantly different. (**C**) Composition of the oropharyngeal flora at the genus level in the two groups. (**D**) Linear discriminate analysis effect size (LEfSe) taxonomic cladogram of oropharyngeal flora differences between T1DM and healthy control children. The hierarchical relationship of all taxa from phylum to genus level is represented by the colored nodes from inner to outer circles. The diameter of the circle = taxa abundance, red = enrichment in T1DM, green = enrichment in healthy controls, and yellow = taxa with non-significant changes. (**E**) The enriched taxa with linear discriminate analysis (LDA). The vertical coordinates in the graph are taxa with significant differences between groups, and the horizontal coordinates are the LDA score values for each taxon. Higher LDA values indicate a higher influence of species richness on variation. Red bars = diabetes group, and blue bars = healthy controls. The LDA threshold was 3, *p < 0.05*. (**F**) Differential gene analysis of the metabolic pathways of oropharyngeal flora in T1DM and healthy control children, blue dots = down-regulation and red dots = up-regulation.

The comparison of oropharyngeal microbiome composition between 13 T1DM and 20 healthy children indicated that the differences in oropharyngeal bacterial diversity (Shannon index, Simpson index) and richness (Sobs index, Chao index) were not statistically significant between the two groups (Figure 2A, Supplementary Figure S1). Dimension reduction methods, including PCoA, indicated no visually discernible differences between T1DM and healthy children (Figure 2B). Next, PLS-DA analysis, a supervised analytical method, was performed, and the bacterial communities in the T1DM samples were clustered separately from those in the healthy control group, indicating the presence of a unique oropharyngeal microbial community between T1DM and healthy control groups (Supplementary Figure S2). PERMANOVA and Wilcoxon rank-sum tests were conducted to calculate the *p*-values (*p* = 0.262 for PERMANOVA and *p* = 0.002 for Wilcoxon), further confirming significant differences in the bacterial communities between the two groups. The oropharyngeal microbiota in T1DM and healthy control groups consisted primarily of *Streptococcus, Prevotella, Leptotrichia*, and *Neisseria* (Figure 2C). Linear discriminant analysis showed that the oropharyngeal bacterial flora of the children with T1DM with *Actinomyces, Achromobacter, F0332*, and *Campylobacter* were significantly enriched (Figures 2D,E, Supplementary Table S1). LEfSe analysis showed that *Actinomyces, Achromobacter, F0332*, and *Campylobacter* could be used as marker bacteria for children with T1DM. The bacteriophage genes or functional units were predicted based on the known microbial genome database KEGG. Volcano plot results showed differences in the metabolic pathways of bacteria in oropharyngeal samples from T1DM and healthy control children (Figure 2F). The top 15 metabolic pathways with the largest differences (LogFC absolute value > 1.5, *p-value < 0.05*) were screened. Significantly down-regulated expression in samples from children with T1DM including PARS, MEXG, NAHAB, MDLB, NAPE, PCHR, HASA, HASR, PARR, ICT-P, ICH-P, OPMD, NORQ, YBTX and FYUA. Among them, the AraC family transcriptional regulator and (S)-mandelate dehydrogenase are involved in the glucose metabolism pathway and phenylalanine metabolism pathway, respectively, and their metabolism was reduced in children with T1DM (Supplementary Table S2).

### 3.3 Microbiome composition of the intestinal tract in children with T1DM and healthy children

**Figure 3:**
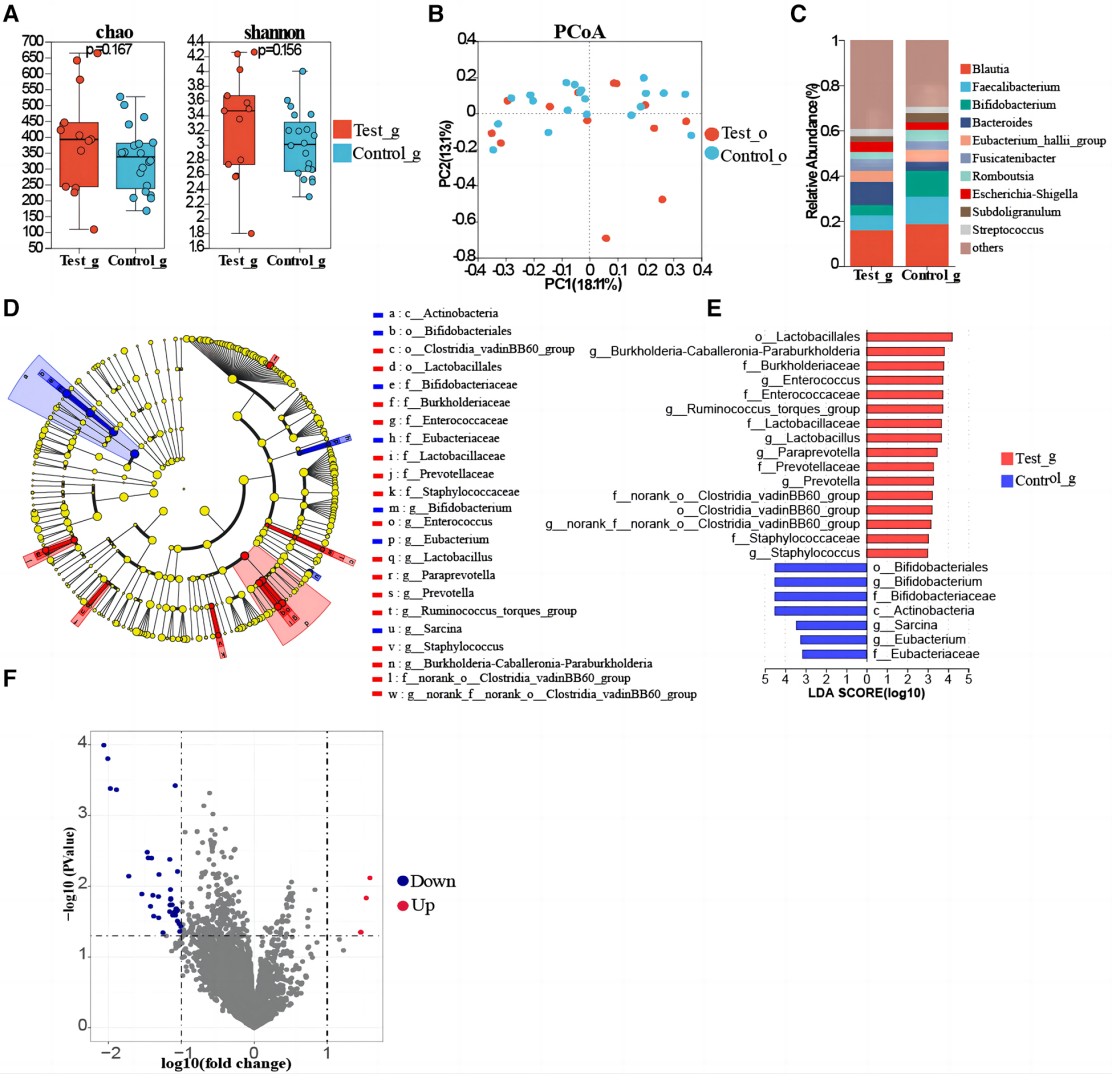
Gut microbial composition of T1DM children. (**A**) Comparison of α-diversity. (**B**) β-diversity analysis of microbiota via PCoA. (**C**) The genus-level composition of the gut flora in the two groups. (**D**) LEfSe study reveals changes in oropharyngeal bacterial flora between T1DM and healthy controls children. (**E**) The bar chart of the LDA discriminant (LDA threshold = 3, *p < 0.05*). (**F**) Differential gene analysis of metabolic pathways of oropharyngeal flora in T1DM and healthy children.

Gut microbial diversity (Shannon index, Simpson index) and richness (Sobs index, Chao index) were not statistically different between T1DM and healthy children (Figure 3A, Supplementary Figure S3). Meanwhile, the PCoA showed no significant difference (Figure 3B). Furthermore, the PLS-DA analysis showed that the bacterial communities in the T1DM samples were clustered separately from the healthy controls, indicating significant differences in the overall structure of the bacterial communities in each group. Moreover, differences between individuals with and without T1DM could also be identified by observing the T1DM samples, which showed a more dispersed distribution pattern compared to controls (Supplementary Figure S4). PERMANOVA and Wilcoxon rank-sum tests were used to calculate the *p*-value (*p* = 0.334 for PERMANOVA and *p* = 2.507e-07 for Wilcoxon), further demonstrating the significant differences between the groups of bacterial communities. At the genus level, the T1DM and healthy control children groups were mainly concentrated in *Blautia, Fusicatenibacter, Bacteroides, Faecalibacterium*, and *Eubacterium_hallii_group* (Figure 3C). Linear discriminant analysis showed that the predominant bacterial groups in the T1DM gut were *Enterococcus, Ruminococcus_torques_group, Lactobacillus, Paraprevotella, Prevotella,* and *Staphylococcus* (Figures 3D,E, Supplementary Table S3). LEfSe analysis showed that *Enterococcus, Ruminococcus_torques_group, Lactobacillus, Paraprevotella, Prevotella*, and *Staphylococcus* could be used as marker bacteria in children with T1DM. The results of the volcano plot showed significant differences in the metabolic pathways of bacteria in gut samples of T1DM and healthy control group children (Figure 3F) and the top 8 metabolic pathways with the largest differences were screened out (LogFC absolute value > 1.5, *p-value < 0.05*). Six metabolic pathways were found to be significantly downregulated in children with T1DM including ADAB, E2.4.2.6, ATL, RBSU, K07498, and CYP152A, while only FUCD and E1.1.1.435 were upregulated. Among them, putative ribose uptake protein and fatty-acid peroxygenase, which are involved in the nucleotide and fatty acid metabolism pathways, respectively, were significantly down-regulated in children with T1DM (Supplementary Table S4).

### 3.4 Correlation between oropharyngeal and intestinal flora in children with T1dm

**Figure 4:**
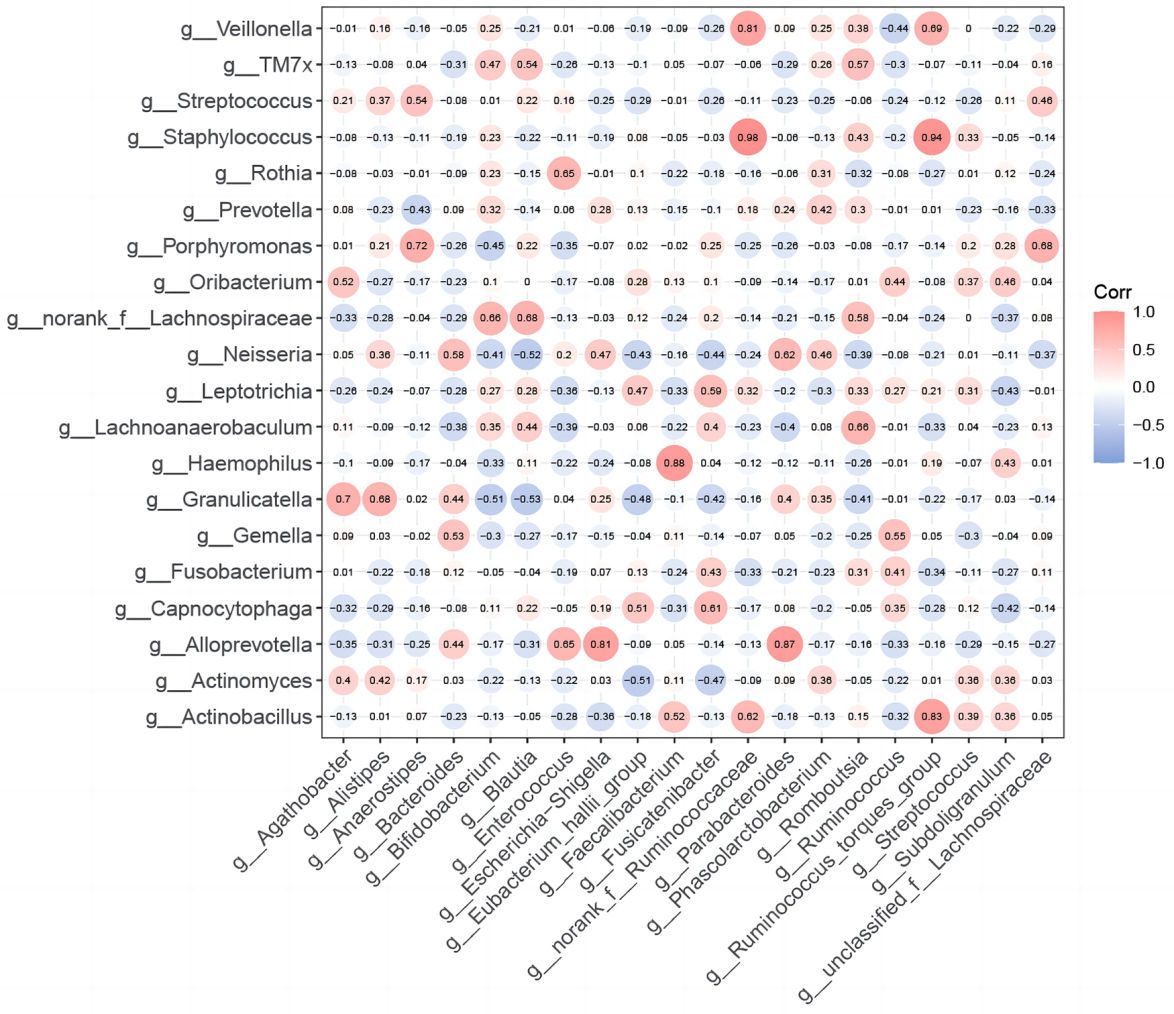
Correlation between oropharyngeal and intestinal flora in children with T1DM. Heatmap of Spearman's correlation coefficients for the top 20 species in the relative abundance of oral and gut microbiota in T1DM children. R-values in the graphs are indicated by different colors. The diameter of each small circle represents the size of the correlation coefficient.

To explore the ecological relationships between different microbial communities and understand potential oropharyngeal-gut microbial relationships, we selected the top 20 species in abundance to draw heatmap diagrams. Between TIDM oropharyngeal and T1DM gut microbiomes: The correlation between dominant bacterial genera was significantly positive according to the (*r* > 0, *p* < 0.05) criterion, and no significant negative correlation was found. (Figure 4). Among them, oropharynx Staphylococcus indicated the highest correlation coefficient with intestinal norank_f__Ruminococcaceae (*r* = 0.98, *p* < 0.001) and Ruminococcus_torques_group (*r* = 0.94, *p* < 0.001). These results preliminarily revealed a potential correlation between oropharyngeal and gut microbiota in children with T1DM. The changes in the relative abundance of oropharyngeal and intestinal flora in children with T1DM had a synergistic effect on each other without a mutual inhibitory effect. Furthermore, this suggests that pathogenic microorganisms in both the oropharynx and intestine of children with T1DM exhibit synchronized changes in disease states.

### 3.5 Correlations between oropharyngeal and intestinal flora and clinical indicators in T1DM children

**Figure 5:**
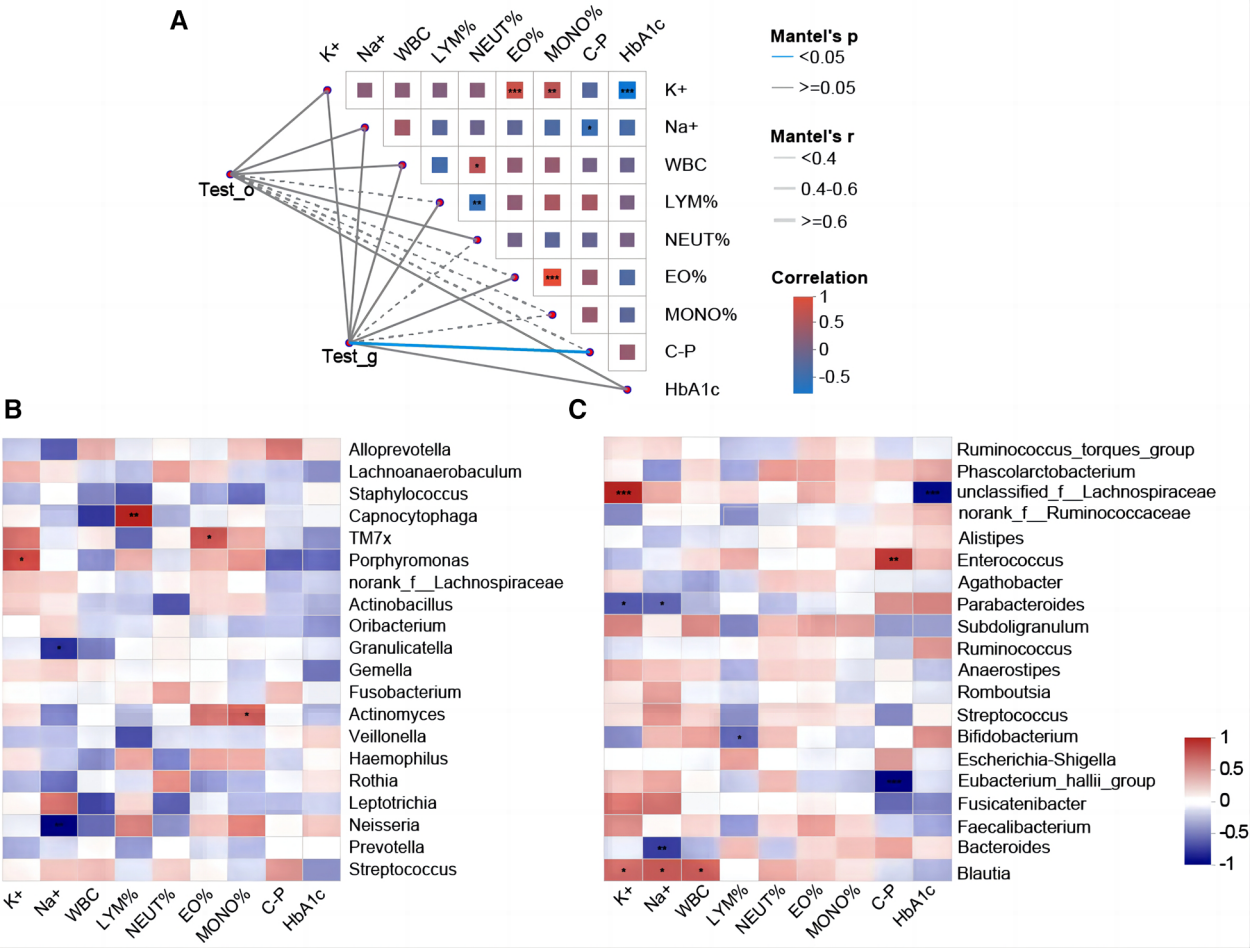
Correlation analysis between oropharyngeal/intestinal flora and clinical indicators. (**A**) T1DM oropharyngeal/intestinal flora correlates with clinical indicators. Mantel-test network heat map (Lines = the correlation between clusters and clinical factors; solid line = positive correlation, and dotted line = negative correlation. The thicker the line, the higher the correlation). Spearman's rank correlation coefficient (r) between the clusters and the clinical factors. The heat map shows the correlation between clinical factors. Red = positive correlation, blue = negative correlation, and an asterisk in the color block = significance. *p ≤ 0.05, ** *p ≤ 0.01*, *** *p ≤ 0.001*. (**B**) Heatmap of the correlation between the top 20 genera of oropharyngeal bacterial flora in terms of their relative abundance and the clinical indicators. Horizontal coordinates = clinical factors, and vertical coordinates = species. The statistical significance was assessed using the Spearman rank correlation coefficients (R) and probabilities (p); R values are shown in different colors, *p < 0.05, **p < 0.01, *** *p ≤ 0.001*. (**C**) Heat map of the correlation between the top 20 species of relative abundance of intestinal flora and clinical indicators.

Spearman correlation analysis revealed a significant positive correlation between gut microbiota species and C-P (*r* = 0.41, *p < 0.05*). Significant negative correlations between HbA1c and K+ and between C-P and Na+ were observed in correlations between several blood indices (Figure 5A). We selected the top 20 bacteria (genus level) in the oropharynx and intestines, respectively, and analyzed their correlation with blood indicators by correlation Heatmap plots. It was found that several bacterial taxa in the oropharynx were significantly correlated with the glycaemic index, with *Alloprevotella* (*r* = 0. 50) and *Streptococcus* (*r* = 0.40) positively correlating with C_P. Furthermore, *Actinomyces* were positively correlated with the percentage of monocytes (MONO%) (*r* = 0.58, respectively), whereas *Capnocytophaga* was positively correlated with the percentage of lymphocytes (LYM%) (*r* = 0.75) (Figure 5B). Additionally, it was found that intestinal *Blautia* (*r* = 0.60), *Bacteroides* (*r* = −0.70), and *Parabacteroides* (*r* = −0.60) showed a significant correlation with Na+. Intestinal *Bifidobacterium* and LYM% (*r* = −0.60, *p < 0.05*), while Intestinal *Blautia* (*r* = 0.60, *p < 0.05*) were significantly correlated with WBC. Moreover, several associations between intestinal microbial taxa and glycaemic indicators were observed: *Eubacterium hallii group* (*r* = −0.91, *p ≤ 0.001*) and *Enterococcus* (*r* = 0.72, *p* *≤* *0.05*) showed a significant correlation with C_P. *Unclassified f_Lachnospiraceae* (*r* = −0.86) and *Fusicatenibacter* (*r* = −0.46) were correlated with HBA1c (Figure 5C).

## 4 Discussion

Much literature indicates that microbiota composition is closely related to T1DM (23). However, there are no reports on the relationship between the oropharyngeal microbiome and T1DM compared to the bacterial community in the oral cavity. Therefore, this study analyzed the microbiome composition of the oropharyngeal and gut ecotopes in TIDM and healthy control children and identified several associations between the bacterial communities of the two ecotopes and clinical indicators.

No significant difference in the α and β diversity of the oropharyngeal microbiome was observed in T1DM children compared to healthy controls, consistent with previous studies (4). In addition, PERMANOVA and the Wilcoxon rank-sum test further confirmed the significant differences between the microbial communities in T1DM and healthy controls, and therefore, oropharyngeal bacterial dysbiosis might be present in T1DM. To the best of our knowledge, this is the first study to show the bacterial composition of the oropharyngeal microbiota in children with T1DM, with the predominant bacteria including *Streptococcus, Prevotella, Leptotrichia*, and *Neisseria*. Streptococci are the earliest bacteria to colonize the human body and are mainly concentrated in the oral cavity (24). Oral *Streptococci* can create acids directly connected to tooth caries development (25). Poor glycaemic control in T1DM patients has been found to promote oral growth of *Streptococcus mutans* (26), higher levels of which have been significantly associated with dental caries (27). There were significant differences in the composition of the oropharyngeal microbiota in T1DM and healthy children. *Actinomyces, F0332, Achromobacter*, and *Campylobacter* indicated significantly higher LDA scores among bacterial taxa in T1DM children, consistent with the literature (28). Furthermore, it was found that different strata of *Campylobacter* were significantly enriched in the oropharynx of T1DM children. *Campylobacter* is a microaerophile that employs pyruvate and 2-oxoglutarate, adapted for growth in the low-oxygen ecological niche of the host, and relies on amino acid metabolism as a major source of energy (29, 30). Glucagon dysregulation in T1DM enhances amino acid metabolism, increasing energy sources and thereby promoting growth. This suggests that *Campylobacter* have a special place in the oropharyngeal microbial community of children with T1DM and may have a broader impact on the development and progression of diabetes.

No significant differences were identified in gut microbial α- and β-diversity compared to healthy controls, consistent with previous studies (31). In addition, PERMANOVA and Wilcoxon rank-sum test further confirmed that the microbial community in T1DM was significantly different than the healthy controls. In T1DM children, the main gut microbes at the genus level included *Blautia, Bacteroides, Fusicatenibacter, Faecalibacterium*, and *Eubacterium_hallii_group. Blautia* regulates lipid metabolism, blood glucose, and T-cell differentiation (32) and its increased abundance can increase intestinal permeability, as well as cause an inflammatory response (33). The *Eubacterium_hallii group* promotes intestinal butyrate and propionate formation (12, 34). Adverse effects on intestinal epithelial barrier function and gut permeability are associated with inflammation (35). Mechanisms such as increased intestinal permeability, inflammatory response, and immune response may play an essential role in the progression of T1DM. These findings suggest that the characteristic gut microbial flora of T1DM patients may play a role in the progression of T1DM. T1DM children have a distinct gut microbiological profile than healthy children and indicated an increased abundance of microorganisms with significantly higher LDA scores, including *Enterococcus, Ruminococcus_torques_group, Lactobacillus, Paraprevotella, Prevotella*, and *Staphylococcus*. It was found that *Ruminococcaceae, Shigella, Enterococcus*, and *Streptococcus* were significantly up-regulated in the T1DM group possibly related to infection and inflammation (36). Some of the bacteria in Ruminococcaceae are pro-inflammatory (37); however, their roles and mechanisms have not been extensively studied. Gram-negative bacilli release lipopolysaccharide (LPS) involved in inflammation in diabetic patients (38), which induces pancreatic β-cell damage (39, 40). These results indicate that dysbiosis is significantly correlated with the incidence of T1DM (41). Therefore, modulating the characteristic gut microbial composition of children with T1DM to reduce intestinal inflammation in diabetic patients may slow disease progression or alleviate diabetic symptoms.

The correlation between oral and intestinal flora alterations has previously been proposed for a wide range of diseases (8, 42). This study revealed that the simultaneous distribution of oropharynx *Staphylococcus* was associated with intestinal *norank_f__Ruminococcaceae* (*r* = 0.98, *p < 0.001)* and *Ruminococcus_torques_group* (*r* = 0.94, *p < 0.001)* with the highest correlation coefficient. Elevated levels of oral *Bacteroides* decreased Th17 cells and an increased M1/M2 macrophage ratio, thereby promoting inflammation in TIDM (43). The literature suggests that changes in oral flora can lead to gut microbiota dysbiosis. *Bacteroides fragilis* aggravates T1DM when intestinal permeability is increased (44). Oral microorganisms (*P. gingivalis* or *F. nucleatum*) increase intestinal mucosal permeability, thus allowing *Bacteroides* to promote the progression of T1DM (45, 46). It was found that oral administration of *Porphyromonas* gingivalis induced changes in the composition of the ileal microbiota, with an increase in the proportion of *Bacteroidetes* phylum, particularly the genus *Bacteroides*, as well as down-regulation of the expression of Sirt1, which induces an increase in glucose uptake and insulin signaling, and that the expression of Sirt1 was negatively correlated with inflammatory genes, especially TNF-α. The changes in the expression of pro- and anti-inflammatory genes may lead to increased serum glucose levels and insulin intolerance (47–49). In this study, the correlation between oropharyngeal and intestinal dominant bacterial genera was significantly positive, and no significant negative correlation was found. Suggests that the changes in the relative abundance of oropharyngeal and intestinal flora in children with T1DM synergize without mutual inhibition. It further indicates that oropharyngeal and intestinal pathogenic microorganisms in children with T1DM show synchronized alterations during disease states, provide evidence for predicting disease trends, and develop appropriate diagnostic and therapeutic strategies based on microbial communities in the oral cavity and gut.

This study analyzed several correlations between oropharyngeal and intestinal flora and clinical indicators in T1DM children. It was revealed that *Actinomyces* were positively correlated with MONO%, and *Capnocytophaga* was positively correlated with LYM%. *Capnocytophaga* is an oral bacterium that often colonizes periodontal tissues and can participate in the development of periodontal disease by modulating the host's immune-inflammatory response. Mononuclear cell counts were most associated with oral microbiota (8). Furthermore, *Bifidobacterium* and *Blautia* were positively correlated with LYM% and WBC, respectively. *Blautia* produces acetate, a metabolite implicated in the regulation of carbohydrate and lipid metabolism, and SCFAs have an impact on host immune regulation and physiological function and play an important role in the development of T1DM (50). There were correlations between HbA1c and K+ and between C-P and Na+ among the blood indices. HbA1c responds to the levels of glycemic control, whereas hyperglycemia and high K+ levels often occur together in diabetic patients. Because of insufficient secretion or impaired action of insulin in diabetic patients, K+ is transferred from the intracellular to the extracellular compartment, causing hyper K+ emia. C-P can be used as a carrier to transport Na+ from outside the cell to the inside to maintain the balance of Na+ inside and outside the cell. In addition, C-P can also act as a signaling molecule involved in intracellular signal transduction processes, in which Na+ is also one of the necessary ions. These correlations have important implications in the diagnosis and management of diabetes, as well as in energy metabolism and signaling. It has been shown that there is a correlation between oral bacteria and the degree of metabolic control of diabetes mellitus (51, 52). This study showed that oropharyngeal *Alloprevotella* and *Streptococcus* were positively associated with positive C_P correlation. Furthermore, *Prevotella* copri, *Alloprevotella* rava, and Ralstonia pickettii abundance were correlated with HbA1c levels in T2DM patients (53). A research study has indicated a correlation between oral *Veillonella* and HbA1c in 89 T1DM children (54). *Veillonella* breaks down carbohydrates and polysaccharides and produces lactic and other organic acids, which can promote the development and progression of dental caries (55, 56). The altered abundance of intestinal flora is significantly correlated with C-P. *Eubacterium_hallii group* (*r* = −0.91, *p < 0.001*) and *unclassified f Lachnospiraceae* (*r* = −0.86, *p < 0.001*) were negatively correlated whereas *Enterococcus* (*r* = 0.74, *p < 0.05*) and *Fusicatenibacter* (*r* = −0.46) were positively correlated with C_P and HBA1c. A study found that pancreatic beta cell function can be improved in diabetic rats by altering gut flora (57), which further validated an association between gut flora and diabetes. However, the causal relationship between gut and C-P is unclear, and further studies are needed to explore this relationship. Furthermore, several small studies have revealed different associations; for instance, a study indicated a negative correlation between *Faecalibacterium* abundance and HbA1c levels in 12 Chinese children with T1DM (58). Another study on 20 Brazilian T1DM children showed a link between the relative abundance of Bacteroidetes, Lactobacillales, and Bacteroides dorei and HbA1c (59). Additionally, an article on 15 Chinese T1DM children discussed the positive correlation between the *Eubacterium_hallii group* and FBG (60). The correlations observed in these studies are not consistent, and further studies integrating multi-omics data are needed to further characterize the potential role of these bacteria on host glycemic control.

This study has several limitations: The number of participants was limited, and the data needs to be confirmed by a large sample cohort study. All the participants were from northern areas of China; therefore, the study does not represent other regions. Multicenter cohort studies are required to confirm the acquired results. Although unknown associations between oropharyngeal and intestinal microbiota and clinical phenotypes in T1DM children were identified, these findings might be insufficient to establish robust associations with clinical phenotypes. Therefore, the study size should be expanded and subjects from different regions should be included. Moreover, the influencing factors should be controlled, and the mechanism of oropharyngeal gut microbial correlation in T1DM should be further explored and validated *in vitro* and *in vivo*.

## 5 Conclusion

Children with T1DM indicated unique microbial characteristics in both the oropharynx and intestines compared to healthy controls. Positive correlations were found between altered relative abundance of oropharyngeal and gut microbiota in children with T1DM. Furthermore, there were associations between oropharyngeal and gut microbiota and with clinical phenotypes. Predicting disease trends by monitoring oral cavity and gut microbial communities provides the basis for further large-scale analyses of these microbial niches-linked mechanisms in children with T1DM. Exploring the relevance of certain oropharyngeal and intestinal microbiomes in TIDM patients to develop new therapeutic strategies for T1DM.

## Data Availability

The datasets presented in this study can be found in online repositories. The names of the repository/repositories and accession number(s) can be found in the article/**Supplementary Material**.
