# Supplementary figures and images for: A pilot study on the characterization and correlation of oropharyngeal and intestinal microbiota in children with type 1 diabetes mellitus
Source: Front Pediatr. 2024 Jun 13;12:1382466. doi: 10.3389/fped.2024.1382466 (PMC11208633; doi:10.3389/fped.2024.1382466)

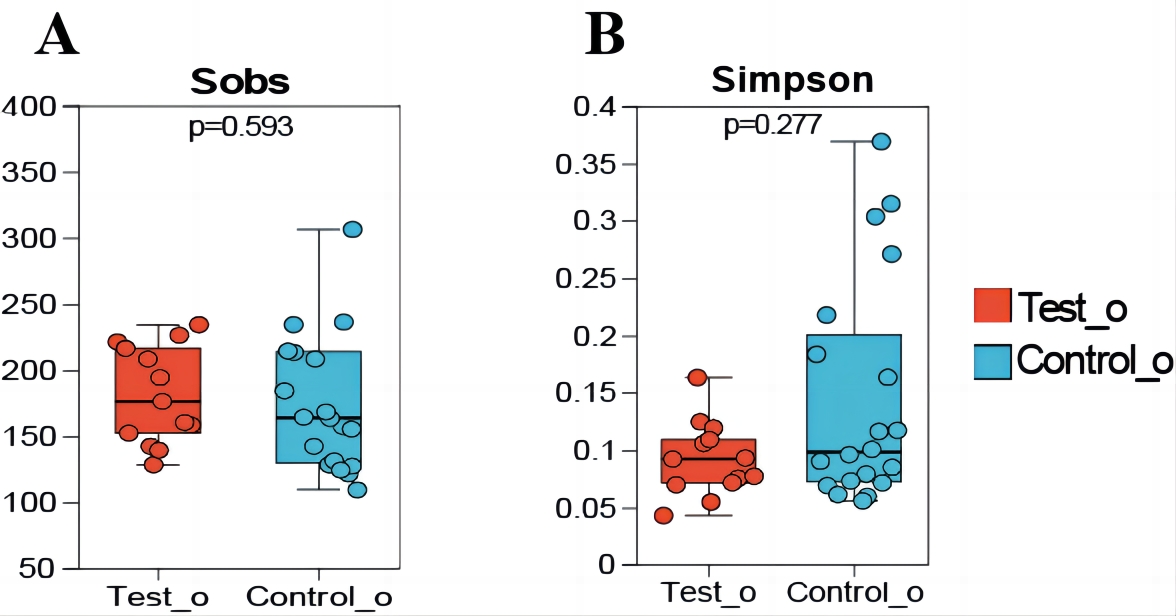

Supplement: Supplementary file 5 [file Image1.jpg]

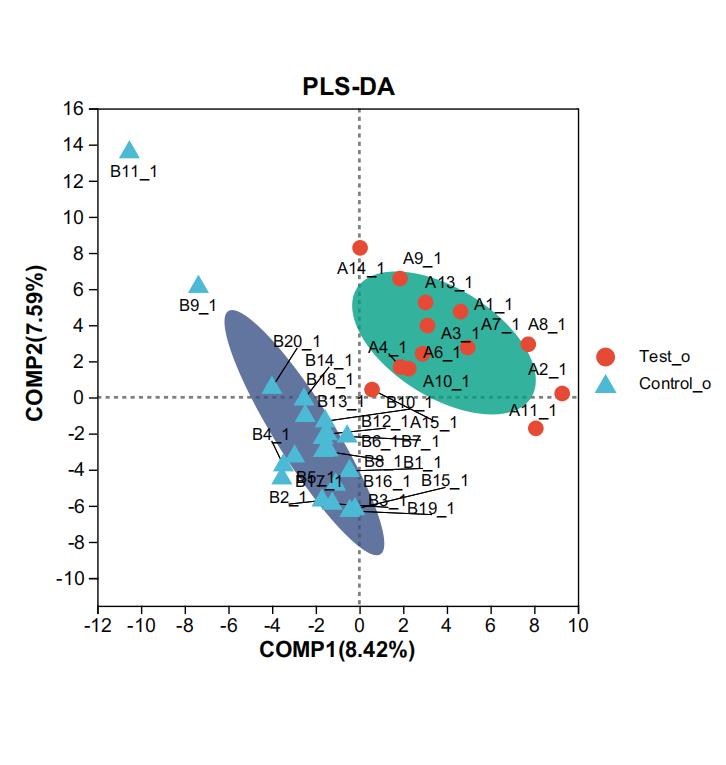

Supplement: Supplementary file 6 [file Image2.jpg]

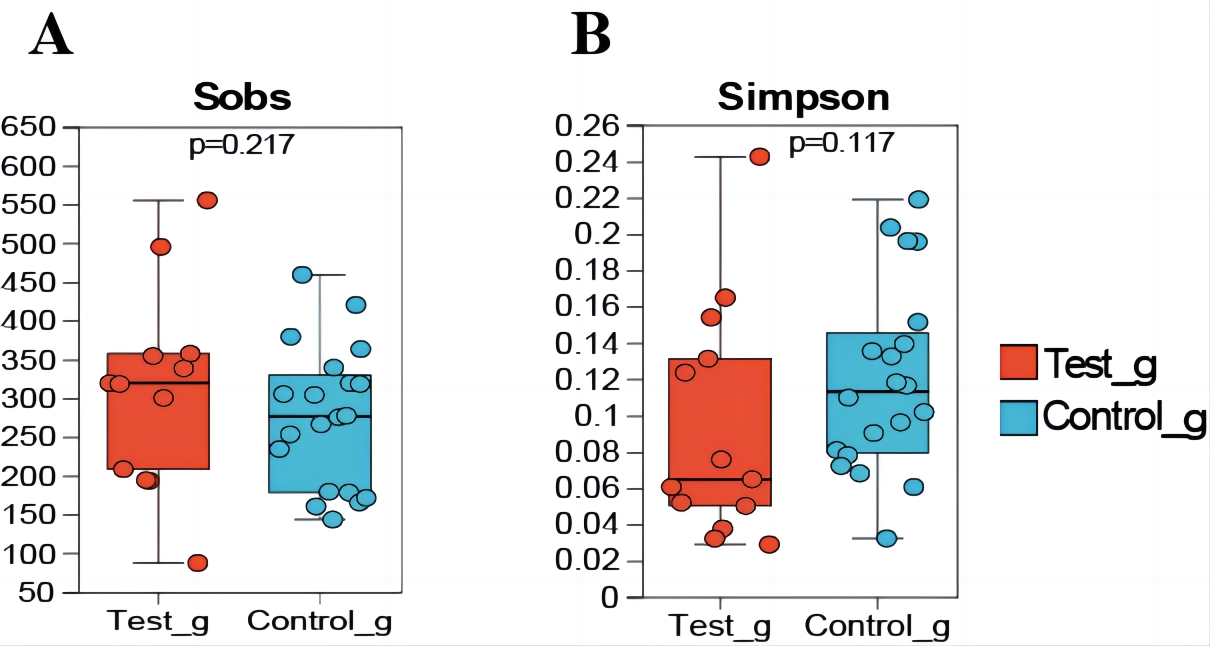

Supplement: Supplementary file 7 [file Image3.jpg]

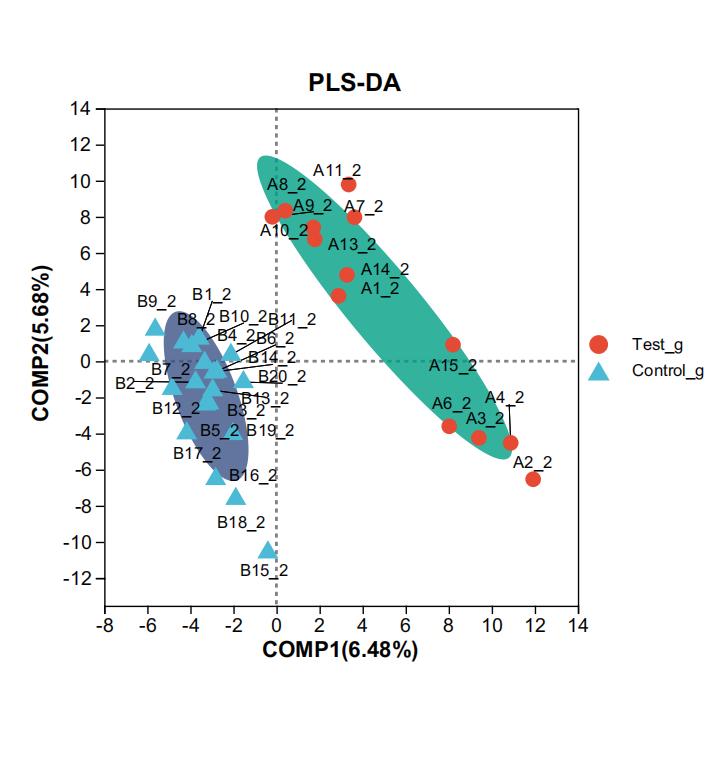

Supplement: Supplementary file 8 [file Image4.jpg]
